# New formulation and approach for mucoadhesive buccal film of rizatriptan benzoate

**DOI:** 10.1007/s40204-017-0077-7

**Published:** 2017-11-06

**Authors:** Sahar Salehi, Soheil Boddohi

**Affiliations:** Department of Biomedical Engineering, Faculty of Chemical Engineering, Tarbiat Modares University, Tehran, Iran

**Keywords:** Mucoadhesive buccal film, Rizatriptan benzoate, Solvent casting, In vitro release, Ex vivo mucoadhesion strength

## Abstract

Mucoadhesive buccal film is developed as a promising dosage form, which has prominent advantages because of drug delivery through buccal mucosa. New formulation of buccal films containing rizatriptan benzoate (RB) was prepared by solvent casting method using various concentrations of hydroxypropyl methylcellulose (HPMC K4M), polyvinyl alcohol (PVA), polyethylene oxide (PEO), glycerol, stevia, and goat buccal mucosa used as a model membrane. In this work, the effect of polymers and plasticizer concentrations on drug release profile, disintegration and dissolution time, mechanical properties, and mucoadhesive characteristics of films was studied. Scanning electron microscopy analysis revealed uniform distribution of RB in film formulations. Chemical compounds and thermal analysis of the films were studied by Fourier transform infrared spectroscopy and differential scanning calorimetry, respectively. The buccal films produced were uniform in drug content and thickness. All formulations have in vitro release of 98–102% between 40 and 80 min. Also ex vivo mucoadhesion strength was in the range of 0.205 ± 0.035 to 0.790 ± 0.014 N for all formulations. A formulation consisting RB (50 mg), HPMC K4M, PVA, and PEO (63 mg), glycerol (1.5 ml), stevia (5 mg) was selected as our optimum composition. More satisfactory results were obtained in terms of disintegration and dissolution time, mechanical properties, and mucoadhesive characteristics. In addition, it showed about 99.89% RB released in 45 min. The results suggest that RB-loaded mucoadhesive buccal films could be a potential candidate to achieve optimum drug release for effective treatment of migraine.

## Introduction

Novel drug delivery systems enhance bioactivity and bioavailability of drugs and reduce their side effects (Lopez et al. 2015). In recent years, mucoadhesive-based drug delivery systems have been widely used in both local and systemic diseases with different applied methods including ocular, nasal, oral, rectal, and vaginal mucosal epithelium (Abd El Azim et al. 2015; Morales et al. 2017).

Oral cavity mucosa is one of the most suitable drug administration routes. This route has many advantages such as preventing drug degradation in gastrointestinal tract, by passing first pass hepatic metabolism, low enzymatic activity, and more patient acceptance compared to ocular, nasal, rectal, and vaginal. It can also permeate low molecular weight drugs through mucosal epithelium quickly because of high surface area of oral mucosal layer when compared to ocular and nasal (Rana and Murthy 2013; Sattar et al. 2014; Silva et al. 2015).

Up today, various dosage forms have been prepared in tablets, patches, gels, ointments, and oral films (Montenegro-Nicolini and Morales 2017; Salamat-Miller et al. 2005). The administration site of mucoadhesive oral drug delivery systems can be sublingual, buccal, gingival, and soft palatal (Borges et al. 2015). Due to high flexibility, minimum side effects, more accurate dosing than drops or syrup formulations, and larger surface area for drug absorption, mucoadhesive buccal films are more preferable than other forms (Khan et al. 2016; Scarpa et al. 2017).

Migraine is a primary headache which causes pulsate, one-sided headache, and common symptoms including vomiting, nausea, and environmental sensitivity which often last 4–48 h (Kristoffersen and Lundqvist 2014). Migraine has four possible phases including prodrome (hours or days before the headache), aura (right before headache), pain (main headache), and postdrome (following effects of headache) (Lauritzen 1994). Nonsteroidal anti-inflammatory drugs including ibuprofen and acetaminophen are effective treatments, but in case of severe and chronic migraine, triptans family such as sumatriptan, eletriptan, zolmitriptan, naratriptan, almotriptan, frovatriptan, and rizatriptan must be used (Gilmore and Michael 2011; Wang et al. 2007).

Rizatriptan benzoate (RB) is the second generation of triptans and 5-HT_1D_ receptor agonist that binds to the endogenous serotonin (5-hydroxy tryptamine) (Visser et al. 1996). RB is often used in migraine treatment because of faster acting than other triptans (Láinez 2006). Although RB is absorbed orally right after administration, studies showed that oral bioavailability is ~ 45% because of extremely hepatic metabolism (Merck and Co. 2013). RB mechanism of action is narrowing brain blood vessels, but it causes few undesirable side effects on cardiovascular system, gastrointestinal tract, respiratory system, and skin. Therefore, the recommended dose of RB in oral dosage form is 5–10 mg per use (Visser et al. 1996). Most patients have experienced and suffered from nausea during the migraine attack, therefore buccal films have been preferred as an appropriate dosage form for the migraine patients (Avachat et al. 2013).

Buccal films generally consist of different ingredients such as polymer, plasticizer, drug, sweetener, and necessary additives. In recent years, polymeric mucoadhesive films have been widely used. They must be non-toxic, biocompatible, and inexpensive and also have an appropriate peel, tensile and shear strength, and sufficient mucoadhesion features. They should not react with the drugs and additives (Krampe et al. 2016).

Polyethylene oxide (PEO)-based buccal films are resistant against fracture and tear. The low molecular weight PEO has a fast dissolution rate in oral saliva. In addition, due to low glass transition temperature, it has been used as a self-plasticizing polymer, which decreases the use of plasticizer in buccal film formulation and enhances loading capacity of drug. On the other hand, both polyvinyl alcohol (PVA) and hydroxypropyl methylcellulose (HPMC K4M) have been widely explored as a film former because of availability in several grades, high tensile strength, good flexibility, and acceptable transparency (Kathpalia and Gupte 2013; Russo et al. 2016).

To date, RB tablets and fast dissolving oral films were developed (Dungarwal and Patil 2016; Vidyadhara et al. 2015). In this study, we aimed to design a new formulation of RB mucoadhesive buccal film and investigated different formulations using HPMC, PVA, PEO, herbal sweetener, and plasticizer to enhance bioavailability.

## Materials and methods

### Materials

RB (purity > 90.0%) was provided by Tehran Chemie Pharmaceutical Co. (Tehran, Iran). Hydroxypropyl methylcellulose (HPMC K4M), polyvinyl alcohol (PVA) (Mw 10000 Da), and stevia were supplied by Sigma-Aldrich (Darmstadt, Germany). Polyethylene oxide (PEO) (Mw 35000 Da) was purchased from Merck Chemical Co (Darmstadt, Germany). Glycerol was obtained by Mojallali Industrial Chemical Complex Co (Tehran, Iran). Disodium hydrogen phosphate (Na_2_HPO_4_), sodium chloride (NaCl), potassium chloride (KCl), potassium dihydrogen phosphate (KH_2_PO_4_), sodium hydrogen carbonate (NaHCO_3_), magnesium chloride (MgCl_2_), calcium chloride (CaCl_2_), and hydrochloric acid (HCL) were purchased from Merck Chemical Co (Darmstadt, Germany) for preparation of phosphate buffer saline (PBS) and Krebs buffer (pH 6.8). Buccal mucosa of goat was purchased from a local slaughter house. Distilled water was used for preparing all aqueous solutions (Zolal, Tehran, Iran). All other ingredients used were of analytical grades.

### Preparation of buccal films

**Fig. 1:**
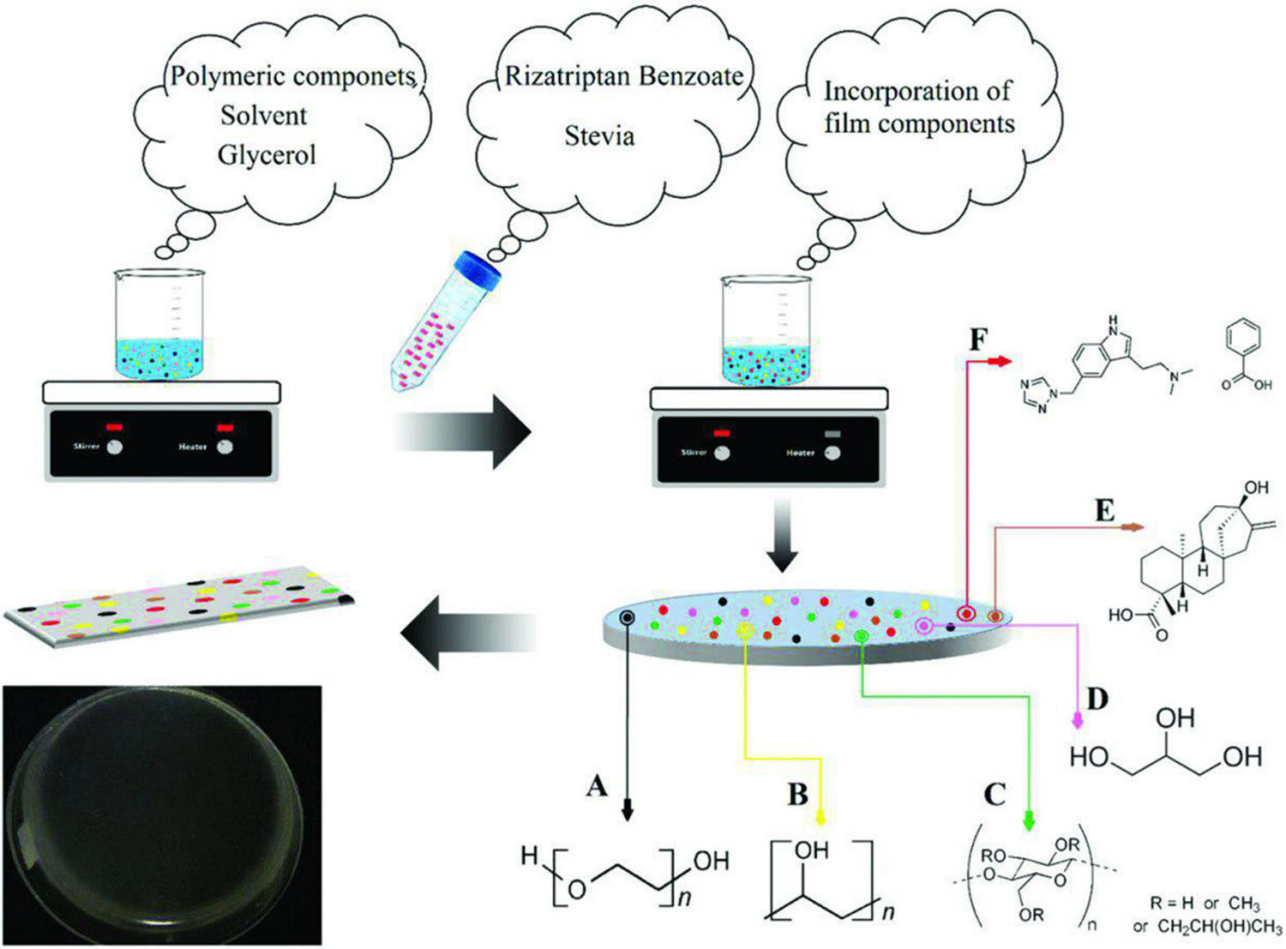
Schematic illustration of buccal film preparation and chemical structure of each component: **a** PEO, **b** PVA, **c** HPMC, **d** Glycerol, **e** Stevia, and **f** RB

Solvent casting method was used to prepare the RB mucoadhesive buccal films because of the simplicity and low-cost operation (Buanz et al. 2015). The preparation procedure is schematically shown in Fig. 1. According to Table 1, different amounts of PVA were dissolved in 10 mL distilled water and stirred for an hour at 400 rpm in 60 °C. Varied amounts of HPMC K4M and PEO were also dissolved separately in 5 mL distilled water and stirred for 15 min at 200 rpm in room temperature. A blend of the above solutions was stirred in 60 °C at 400 rpm for an hour with continuous addition of glycerol as a plasticizer. In this study, PEO was used as both self-plasticizing polymer and compatibilizer in the polymeric mixture. The film structure is depicted in Fig. 1 which shows good compatibility with both HPMC K4M and PVA.

**Table 1:** Different formulations of RB mucoadhesive buccal films

Formulations	PEO (mg)	PVA (mg)	HPMC (mg)	Glycerol (ml)	RB (mg)	Stevia (mg)
F1	63	63	63	1.5	50	5
F2	75	75	75	0.5	50	5
F3	84	63	63	1.5	50	5
F4	63	75	63	2	50	5
F5	63	42	63	1.5	50	5
F6	63	84	63	1.5	50	5

Finally, stevia as a sweetener and RB were poured into the latter solution and stirred for 3 h at 400 rpm in room temperature until the opaque suspension was changed to clear viscous solution. The solution was poured into the 35-cm^2^ petri dish and kept at 40 °C for 48 h in the oven for solvent evaporation. The resulting films were cut to 6 cm^2^ pieces by a cutter for further analysis. In this study, six formulations of RB-loaded mucoadhesive films were prepared. The detailed description of six formulations (F1–F6) is given in Table 1. All formulations were analyzed for physical properties, degradation rate, drug content uniformity, mucoadhesion strength, and in vitro release study. Each measurement was repeated twice and average values were reported. In addition, physicochemical properties of optimized films were evaluated by scanning electron microscopy (SEM), differential scanning calorimetry (DSC), and Fourier transform infrared spectroscopy (FTIR).

## Characterization of buccal films

### Scanning electron microscopy (SEM)

Sample solution (1 mL) was deposited on a glass chip. Samples were dried in the oven (40 °C) under vacuum before further SEM experiments. Dried solution of RB, film polymers formulation without RB, and buccal film containing RB were sputter coated and imaged by scanning electron microscope (Philips, Netherlands) at operating voltage of 10–20 kV and magnification of 500X–5000X.

### Fourier transform infrared (FTIR) spectroscopy

In FTIR spectrometer (Frontier PerkinElmer, Waltham, MA, USA), 10 mg of RB and freeze-dried buccal film formulation with and without RB were used to investigate possible chemical interactions between RB and other components of film formulation. Each sample was scanned in wavenumber range of 400–4000 cm^−1^ at resolution of 2 cm^−1^. The resulting spectrum was analyzed by Perkin Elmer Spectrum v10.03.06 software (PerkinElmer, Waltham, MA, USA).

### Differential scanning calorimeter (DSC)

DSC (Netzsch, Selb/Bavaria, Germany) was performed for possible crystal rearrangement of RB with film components. About 5 mg of each sample was put into an aluminum pan and all scans were carried out under nitrogen stream (15 mL/min). The resulting curves were obtained from 0 to 300 °C in an average heating rate of 10 °C/min.

### Surface pH

The surface pH of all formulations was determined to check whether each film causes irritation to the buccal mucosa. To measure the surface pH of RB films, they were kept in 5 mL distilled water for 10 min to swell. After complete swelling, the surface pH was measured by pH meter (Equip-Tronics, EQ-612, India). pH probe was in contact with the surface of each film and was allowed to equilibrate for 1 min. The average values are reported in Table 2.

**Table 2:** Surface pH, thickness, drug content and folding endurance of different film formulations

Formulation	Surface pH	Thickness (mm)	Drug content (%)
F1	6.89 ± 0.04	0.174 ± 0.010	98.39 ± 0.58
F2	6.95 ± 0.05	0.284 ± 0.005	100.98 ± 1.84
F3	6.54 ± 0.03	0.277 ± 0.025	101.87 ± 1.14
F4	6.92 ± 0.07	0.265 ± 0.036	102.70 ± 1.61
F5	6.63 ± 0.06	0.168 ± 0.011	99.29 ± 0.53
F6	6.75 ± 0.08	0.274 ± 0.013	98.86 ± 0.81

### Buccal film thickness

The film thickness is an essential factor that exhibits the distribution uniformity of formulation components. Film thickness was measured using a digital micrometer (293 MDC-MX, Mitutoyo Co., Kawasaki, Japan) in five different regions of each formulation. Maximum difference among all five regions in film should be less than 5% (Karki et al. 2016). The average values were used for further investigation.

### Folding endurance

Folding endurance was assessed by repeatedly folding specified region of each film (3 × 2 cm^2^) at the same point until breaking occurs. Number of times a film was folded without breaking was reported as folding endurance value (Mahesh et al. 2010).

### Swelling ratio (%)

After calculating the primary weight of 3 × 2 cm^2^ film (W_1_), the swelling properties of films was determined by placing films in PBS (pH 6.8) at 37 °C. At specified time intervals of 5 min, films were removed from PBS solution and excess PBS was removed with filter paper until the films degraded. The swollen films were weighed (*W*_2_) and swelling ratio was calculated using following equation (Peh and Wong 1999; Shiledar et al. 2014):

1 $$ S(\% ) = \frac{{(w_{1} - w_{2} )}}{{w_{1} }} \times 100. $$

### In vitro disintegration and dissolution time study

In vitro disintegration and dissolution time were determined by the same method described in the United States Pharmacopeia (USP) (Irfan et al. 2016). Films were cut into 3 × 2 cm^2^ segments and put in a petri dish with 15 mL PBS (pH 6.8). Then they were placed in incubator shaker (SI-300, Incubator-Shaker, Osaka, Japan) at 37 °C with rotation speed of 50 rpm. In vitro disintegration time was measured when films started to break and the dissolution time was measured when they were completely dissolved in PBS.

### Tensile strength and percentage elongation

Santam testing machine (STM-20, Santam LTD., Tehran, Iran) was used to calculate tensile strength (TS) and percentage elongation-at-break (%EB) of the films. TS is the maximum stress applied to specified part of films without tearing. EB% is the maximum deformation of films length without tearing. Film (*L*_0_ initial length, *t* thickness, *w* width) was placed between the clamps lever of instrument, and an extension force at the speed of 2 mm/min was applied to each film. At tearing time, load at failure (*F*) and final length (*L*) was measured. TS and EB% were calculated using following equations (Dixit and Puthli 2009):

2 $$ {\text{TS}}\;\left( {\frac{N}{{{\text{cm}}^{2} }}} \right) = F \times \frac{100}{t \times w}$$

3 $$ {\text{EB}}\%\;({\text{cm}}\% ) = \frac{{\left( {\left( {L - L_{0} } \right) \times 100} \right)}}{{L_{0} }}. $$

### Young’s modulus

Young’s modulus or elastic modulus is calculated for measuring film stiffness. It is defined by applied stress over strain ratio in the elastic deformation zone. Hard and brittle films represent high TS and low elongation; therefore, these films have high Young’s modulus. Young’s modulus was calculated using following equation (Dixit and Puthli 2009):

4 $$ {\text{Young's}}\,{\text{modulus}} = \frac{{\left( {{\text{slope}}\,{\text{of}}\,{\text{stress}}\,{\text{vs}}\,{\text{strain}}\,{\text{plot}} \times 100} \right)}}{{\left( {{\text{film}}\,{\text{thickness}} \times {\text{crosshead}}\,{\text{speed}}} \right)}}. $$

### Ex vivo mucoadhesive strength

In this study, buccal mucosa of goat was freshly cut as a model for measuring the mucoadhesion strength of film. To avoid being rotten, a piece of buccal mucosa was kept in Krebs buffer and stored at 4 °C for 2 h. Goat mucosa reached room temperature before further use. Films adhered to the upper lever of Santam instrument (STM-20, Santam LTD., Tehran, Iran) and goat mucosa was adhered to the fixed lever by a few drops of PBS (pH 6.8). The film was in contact with mucosa for 1 min. Afterward, the movable lever moved up at speed of 2 mm/min. The force required to tear apart the mucosal surface was calculated and reported as the mucoadhesive strength of the film (Bahri-Najafiet al. 2014).

### Ex vivo residence time

To obtain film residence time on goat mucosa surface, agitators Bain-Marie (GFL-1086, GFL, Burgwedel, Germany) was used at rotating speed of 50 rpm at 37 °C. A 3 × 2 cm^2^ piece of film was placed on the outer layer of mucosa, and both layers were put into the petri dish filled with 5 mL PBS (pH 6.8). Afterward, they were put in the Bain-Marie. Residence time is the time when film is disintegrated on buccal mucosa.

### Drug content uniformity

**Fig. 2:**
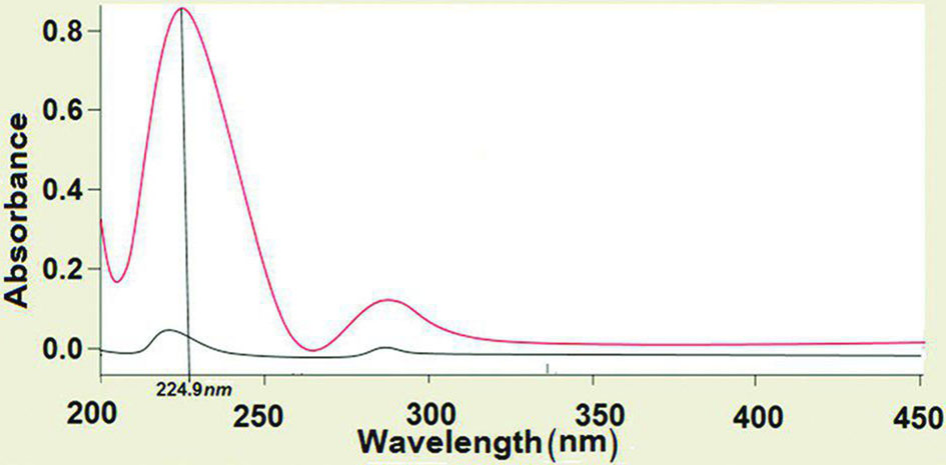
UV spectra of RB in PBS (pH = 6.8)

**Fig. 3:**
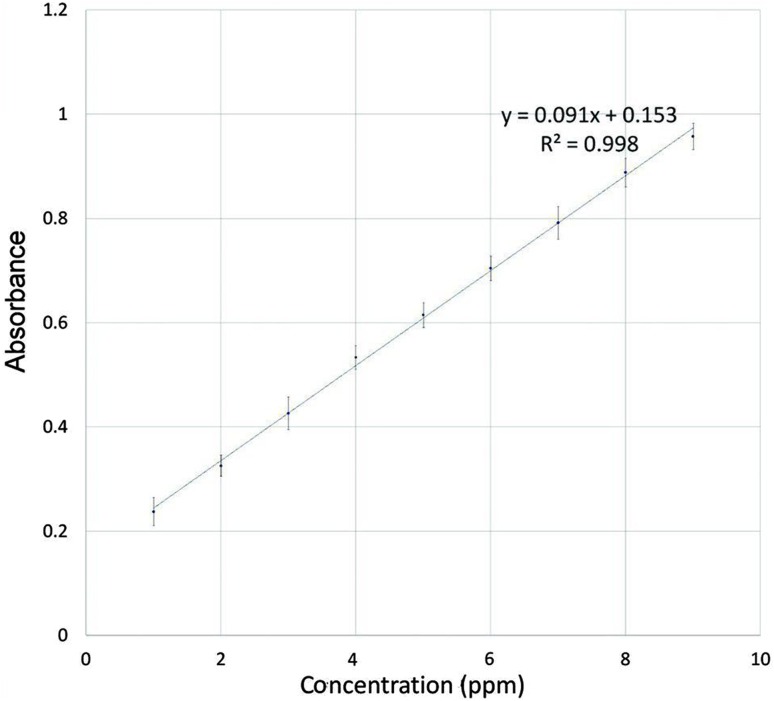
Standard curve of RB in PBS (pH = 6.8)

Films were cut into 3 × 2 cm^2^ pieces and placed in a petri dish filled with 250 mL PBS (pH 6.8). Agitators Bain-Marie was used for complete dissolution of the film in PBS at rotating speed of 50 rpm at 37 °C. The UV–Vis spectrophotometry (Cary 50-Conc, Varian, California, USA) was used to measure the actual amount of RB in each film at a wavelength of 224.9 nm. UV spectrum of RB is depicted in Fig. 2. Also, standard curve was obtained in PBS (pH 6.8) (Fig. 3). Limit of uniformity of drug content is 85–115%. This parameter was calculated according to the following equation (Chaudhary et al. 2013; U.S. Pharmacopeia 2011):

5 $$ {\text{RB}}\,{\text{content}}\,{\text{uniformity}} = \frac{{\left( {{\text{actual}}\,{\text{amount}}\,{\text{of}}\,{\text{RB}}\,{\text{in}}\,{\text{film}} \times 100} \right)}}{{{\text{theoretical}}\,{\text{amount}}\,{\text{of}}\,{\text{RB}}\,{\text{in}}\,{\text{film}}}}. $$

### In vitro drug release

In vitro drug release studies were carried out using agitators Bain-Marie at rotating speed of 50 rpm and 250 mL of PBS (pH 6.8) at 37 °C. In order to study RB release, films were cut into 3 × 2 cm^2^ pieces and moved to an Erlenmeyer flask containing 250 mL PBS (pH 6.8), and were placed in Bain-Marie. At predetermined time intervals of 5 min, 1 mL of release media was withdrawn and passed through 0.45-µm filter. The concentration of RB released from buccal film was estimated using UV–Vis spectrophotometer at 224.9 nm. The release medium was replaced with 1 mL of fresh PBS to maintain constant volume.

### Drug release mechanisms

RB release kinetic was evaluated based on four mathematical models: zero order, first order, Higuchi, and Korsmeyer–Peppas (Mathiowitz 1999; Siepmann et al. 2011).

In the zero-order release model, drug release rate is independent of concentration. Dosage forms following this model are ideal systems.

6 $$ Q_{t} = Q_{0} + \left( {K \times t} \right) $$

In this case, *Q*_0_ is the initial amount of drug released, *Q*_*t*_ is the cumulative amount of drug released at time *t*, and *K*_0_ is zero-order release model constant.

In first-order release model, release rate depends directly on the amount of remaining drug in film. Therefore, release rate decreases over time due to the reduction of remaining drug.

7 $$ {\text{Log}}\,Q_{t} = {\text{Log}}\,Q_{0} + \left( {\frac{{K_{1} \times t}}{2.303}} \right) $$

In this case, *K*_1_ is the first-order release model constant.

Higuchi release model is developed for matrix-based drug delivery systems following diffusion-controlled release.

8 $$ Q_{t} = K_{\text{H}} \times t^{1/2} $$

In this case, *K*_H_ is the Higuchi release model constant.

Korsmeyer et al. described the dependence of drug release with different polymeric carriers. To derive the primary release mechanism, only first 60% of drug released data were fitted to Korsmeyer–Peppas equation.

9 $$ {\text{Log}}\left( {Q_{t} /Q_{\infty } } \right) = \log \,K_{p} + \left( {n \times {\text{Log}}\,t} \right) $$

In this case, *Q*_∞_ is the total amount of drug released in dosage from, *K*_P_ is Korsmeyer–Peppas release model constant, and *n* is the release model exponent, which depends on mass transfer mechanism of the drug. For the case of film drug delivery systems, *n* = 0.5 corresponds to a Fickian diffusion mechanism, 0.5 < *n* < 1 corresponds to anomalous transport mechanism, and *n* = 1 refer to polymer swelling mechanism.

## Results and discussion

### Morphological analysis

**Fig. 4:**
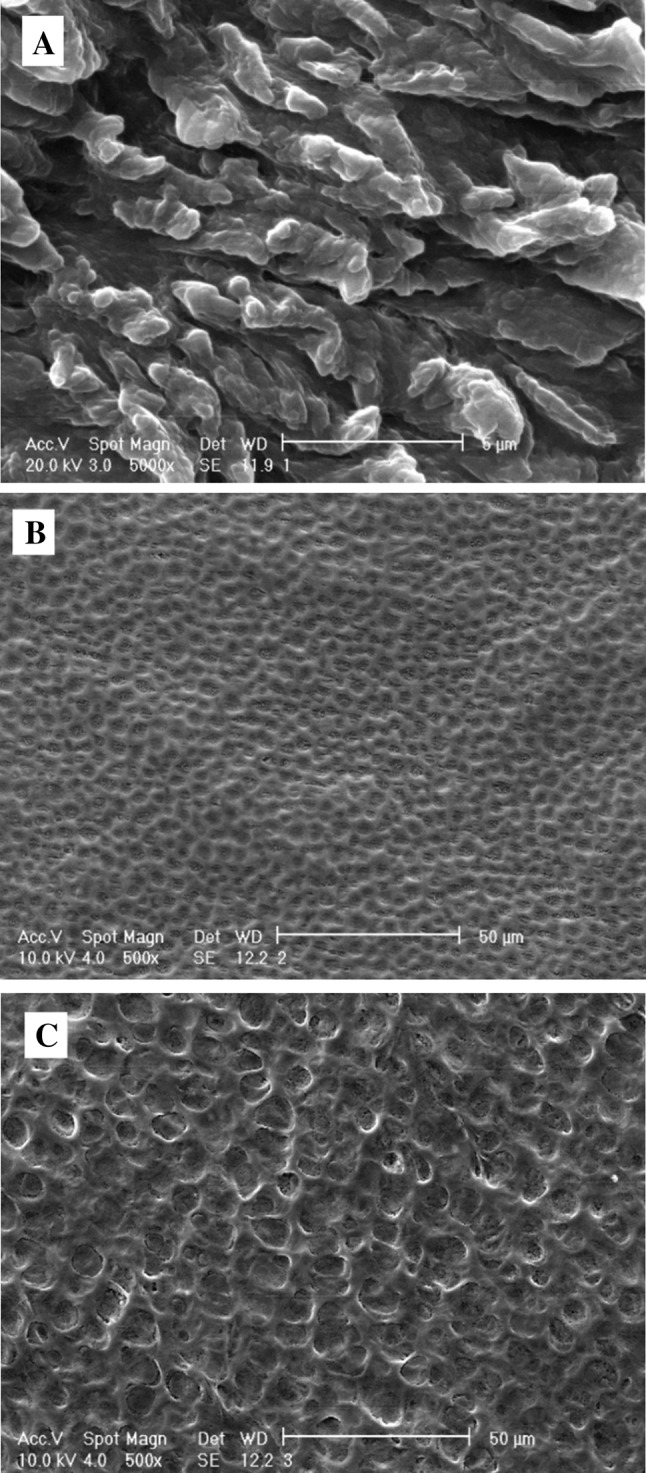
SEM image of **a** RB drug, **b** film formulation, and **c** buccal films with RB

The surface morphology of RB, film without RB, and film containing RB were analyzed by SEM and are shown in Fig. 4. Representative scanning electron micrograph of only RB sample shows regular crystal structure with a size range of 3–5 µm (Fig. 4a). Film without RB shows uniform structure with small pores and average size of 5 µm (Fig. 4b). In film containing RB, scanning electron micrograph depicts uniform surface with bigger pore size and an average size of 10 µm (Fig. 4c). Results indicate that the images of film formulation with and without RB have still unvaried appearance on surface.

### FTIR spectroscopy measurement

**Fig. 5:**
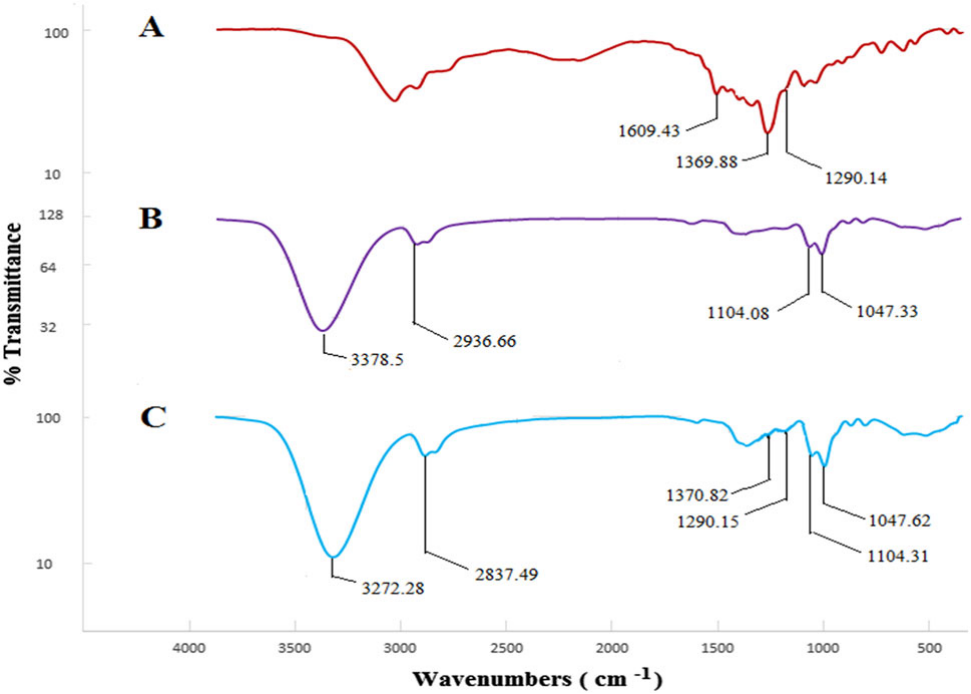
FTIR spectra of **a** RB drug, **b** film formulation, and **c** buccal films with RB

FTIR was performed to detect possible interactions between RB and other components of buccal film formulation (HPMC, PVA, PEO, glycerol, and stevia). FTIR spectra for RB show peak at 1609 cm^−1^ which is assigned to C=C stretching in aromatic rings. The important absorption peak at 1370 cm^−1^ is attributed to C–N stretching in tertiary amines. The corresponding peak at 1290 cm^−1^ is observed for C–O stretching in carboxylic acid (Fig. 5a) (Dungarwal and Patil 2016; Vidyadhara et al. 2015). Figure 5b shows spectra for components of buccal film formulation (a film without RB). Peak at 3379 cm^−1^ is observed for O–H stretching in hydroxyl group. The peak at 2937 cm^−1^ was attributed to C–H stretching vibration in an alkane. The peaks at 1048 and 1104 cm^−1^ are assigned to C–O stretching in hydroxyl group (Bianchi et al. 2011; Rustemkyzy et al. 2015). Figure 5c shows the FTIR spectrum of film containing RB with optimized formulation. There are no new peaks generated and no significant peak shifts are observed although there might be possible interaction between drug and film components.

### DSC analysis

**Fig. 6:**
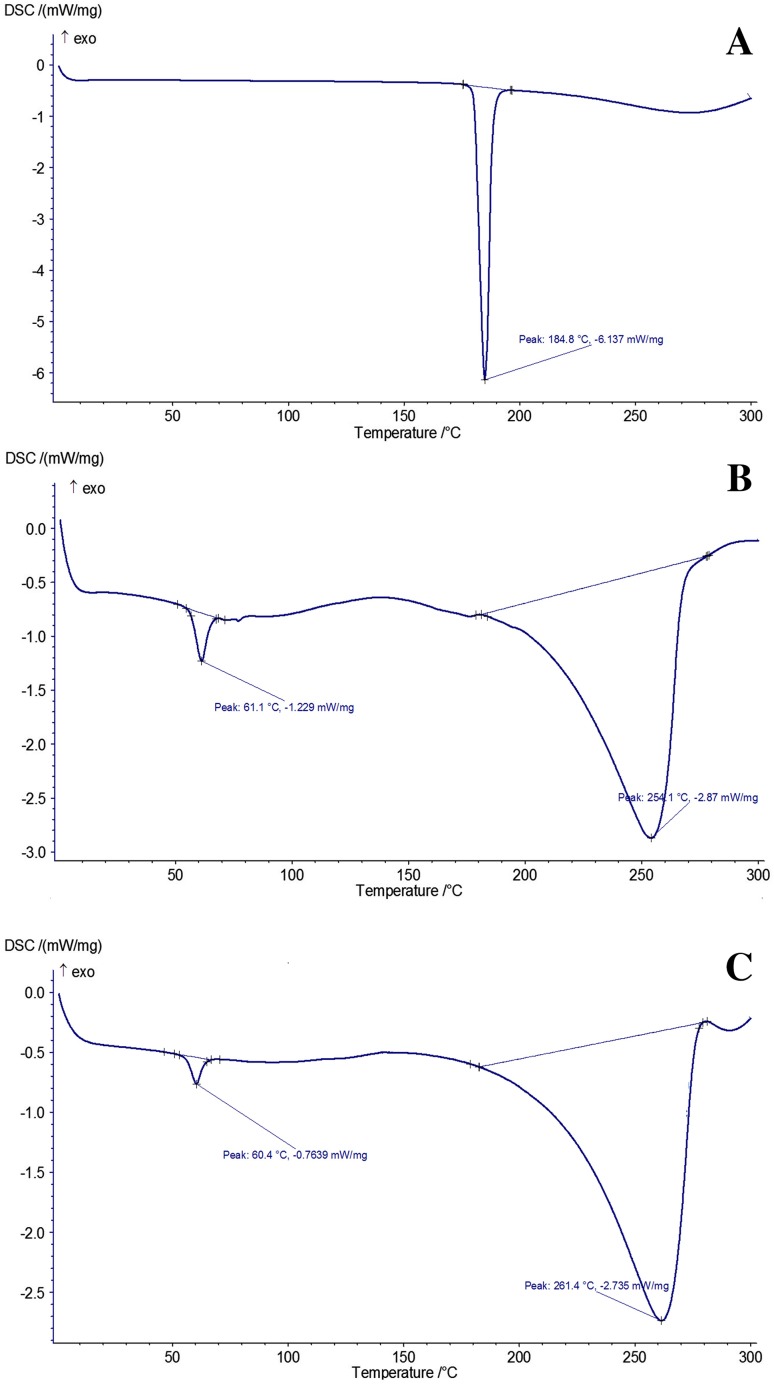
DSC thermogram of **a** RB drug, **b** film formulation, and **c** buccal films with RB

DSC thermograms of RB, buccal film formulation components with and without RB are shown in Fig. 6. DSC thermogram of RB exhibits a sharp endothermic peak at 184.8 °C, which corresponds to its melting point (Fig. 6a). DSC thermogram of other components in film formulation (a film without RB) is shown in Fig. 6b. The first small peak at 61.1 °C could be assigned to dehydration and the second large endothermic peak at 278.8°C corresponded to melting process. In Fig. 6b, no separate characteristic melting peak is observed for each component which could result in compatibility of materials with each other in film structure. Figure 6c shows DSC thermogram of film formulation components with RB, which have slight difference compared to film without RB. In addition, the endothermic peak of RB is completely disappeared due to the incorporation of drug within the film components. Results indicate compatibility between RB and other components of film formulation which also confirm FTIR results (Dungarwal and Patil 2016; Vidyadhara et al. 2015).

### Surface pH

The effect of pH on buccal mucosa was examined by measuring surface pH of films. The surface pH of RB films was measured in the range of 6.54 ± 0.04 to 6.98 ± 0.01 for all formulations (Table 2). Results show that surface pH of films are in the range of healthy human saliva, which is 6.3–7.3 (Aframian et al. 2006).

### Thickness and drug content uniformity

Uniformity of thickness and drug content are related to the accuracy of drug concentration in different regions of each film (Dixit and Puthli 2009). All measurements were replicated and the film thickness was found to be in the range of 0.168 ± 0.011 to 0.284 ± 0.005 mm. In addition, drug content uniformity percentage was determined in the range of 98.86 ± 0.81 to 102.70 ± 1.61%. Uniformity in thickness and drug content of all formulations are shown in Table 2. Results indicate that buccal films are uniform due to the negligible and acceptable changes in thickness and drug content.

### Folding endurance

Folding endurance was measured to determine rupturing resistance and mechanical strength of buccal films. When folding endurance increased, mechanical strength increased as well. The folding endurance of all formulations was measured over 100 times, which showed good flexibility (Liew et al. 2012).

### Swelling ratio

**Fig. 7:**
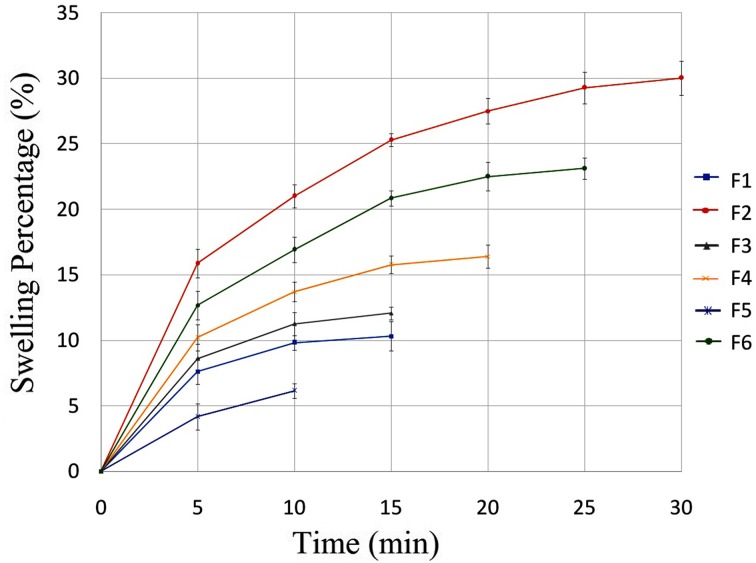
Swelling ratio of six buccal film formulations

Swelling ratio of all formulations is shown in Fig. 7. Swelling ratio of each film was measured until film was degraded. Therefore, because of various components in each formulation, swelling ratio was measured at different time points. F2 formulation, which contained the highest concentration of HPMC K4M, showed highest swelling ratio (30%) up to 30 min. However, among all formulations prepared with constant HPMC K4M concentrations, F6 formulation which contained higher concentration of PVA, possessed more swelling ratio. Based on Table 1, formulations 1 and 3 have different PEO concentrations but swelling ratios of both formulations are almost equal in 15 min. Therefore, results show that different concentrations of PEO do not change swelling ratio significantly.

### In vitro disintegration and dissolution time

**Fig. 8:**
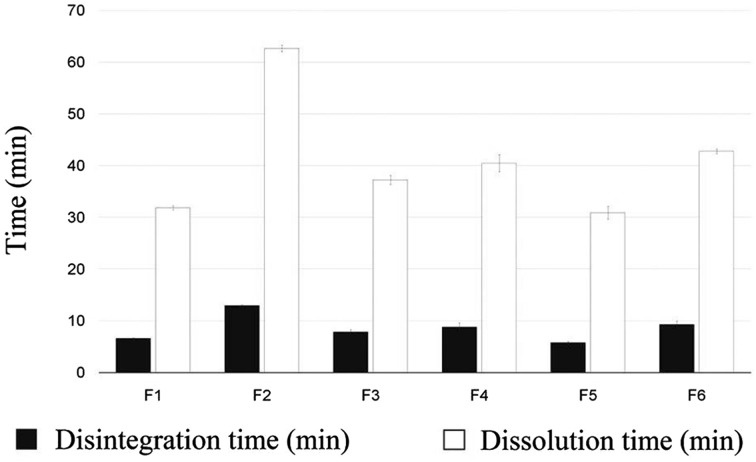
Comparative evaluation of disintegration and dissolution time of six buccal film formulations

In vitro disintegration time and in vitro dissolution time were measured twice and were approximately in the range of 5.80 ± 0.24 to 12.93 ± 0.14 min and 30.89 ± 1.26 to 62.65 ± 0.61 min, respectively. Results of in vitro disintegration and dissolution time of all formulations are shown in Fig. 8. As we expected, increment in polymer concentration and viscosity could enhance disintegration and dissolution time. In addition, in vitro disintegration and dissolution time increased with higher amount of HPMC K4M, PVA, and PEO in all formulations.

### Tensile strength, percentage elongation, and Young’s modulus

**Fig. 9:**
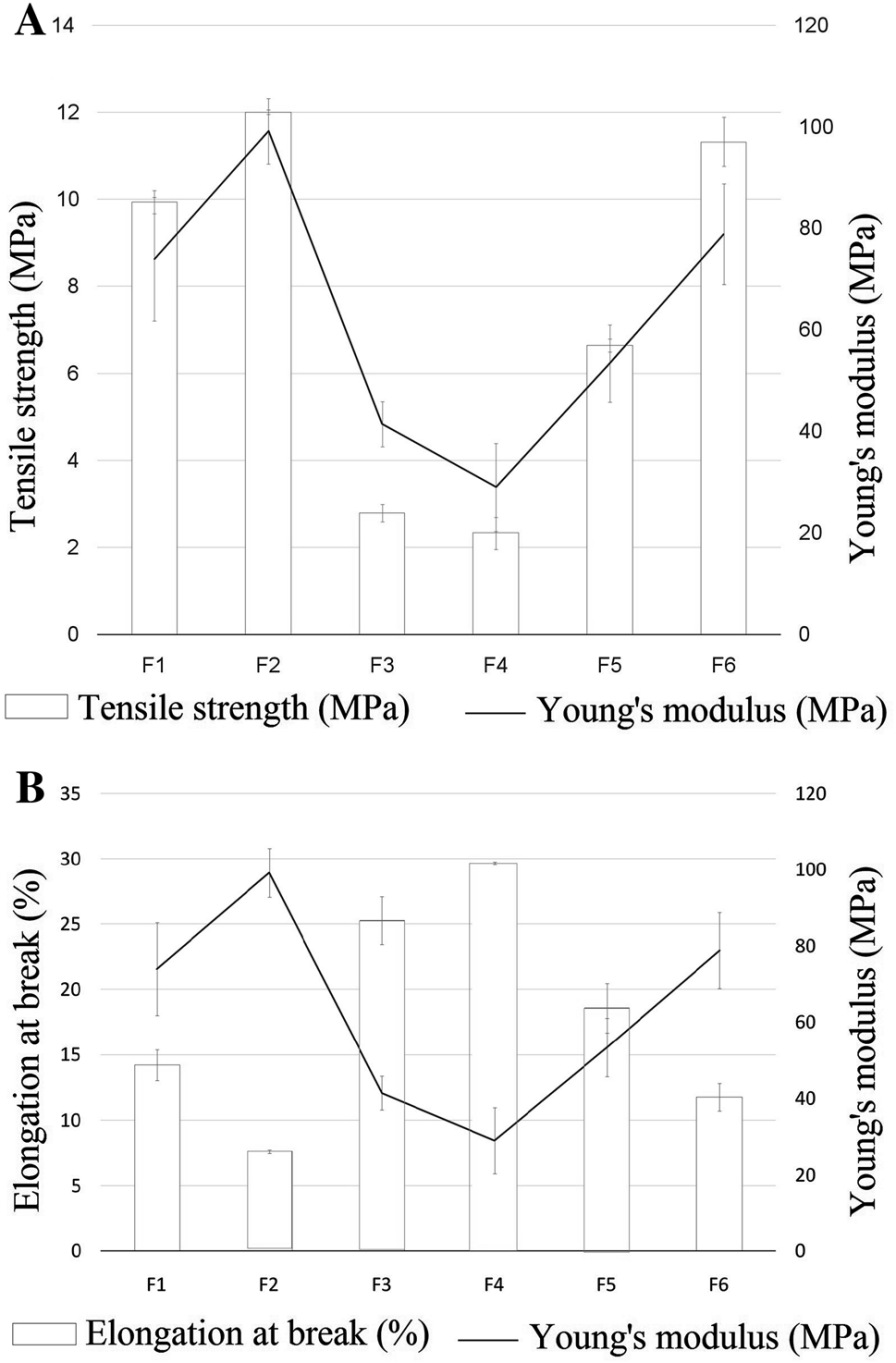
Comparative evaluation of mechanical properties of six buccal film formulations: **a** combined illustration of tensile strength and Young’s modulus; **b** combined illustration of elongation-at-break and Young’s modulus

Tensile strength and percentage elongation varied with different concentrations of polymers. The results of tensile strength and percentage elongation of all formulations are shown in Fig. 9. Tensile strength was measured in the range of 2.33 ± 0.37 to 12.01 ± 0.049 MPa. Percentage elongation was found in the range of 7.61 ± 0.117 to 29.66 ± 0.094%. F2 formulation showed higher tensile strength and lower percentage elongation than the other formulations, but F5 formulation showed higher percentage elongation and lower tensile strength. Results showed that tensile strength increased with increasing HPMC K4M and PVA concentration. In addition, the percentage elongation enhanced with increasing amount of PEO and glycerin in the formulations. Young’s modulus experiments showed when films were harder, Young’s modulus and tensile strength increased and percentage elongation decreased.

### Ex vivo mucoadhesive strength and residence time

Mucoadhesive strength and residence time varied with different concentrations of polymers. Residence time was found in the range of 11–18 min. Basically, disintegration of each film on buccal mucosa occured at specific residence time which is shown in Table 3. The mucoadhesive strength was also observed within the range of 0.205 ± 0.035 to 0.790 ± 0.014 N. F2 and F5 formulations showed the highest and the lowest mucoadhesive strength and residence time, respectively, among other formulations due to HPMC K4M content in the formulations (Table 3). Therefore, HPMC K4M increased mucoadhesive strength and residence time because of higher viscosity grade than PVA and PEO (Shanker et al. 2009).

**Table 3:**
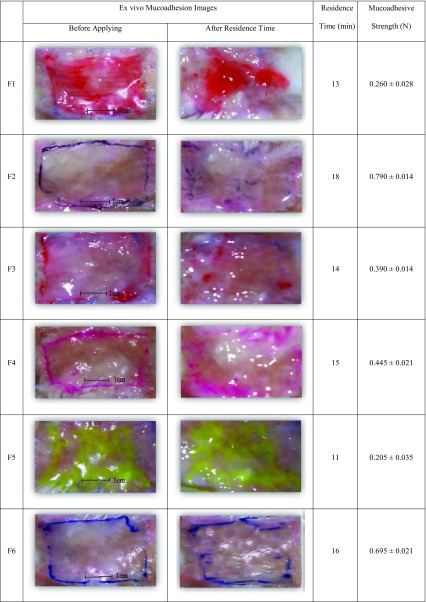
Ex vivo mucoadhesion study of different formulations of buccal films

Overall, results in Table 3 and Fig. 7 illustrate that ex vivo mucoadhesive strength and residence time of all formulations could depend on swelling ratio of each film. Many research groups also described that there were direct relationship between swelling ratio and adhesion force (Adhikari et al. 2010; Patel et al. 2007).

### In vitro drug release

**Fig. 10:**
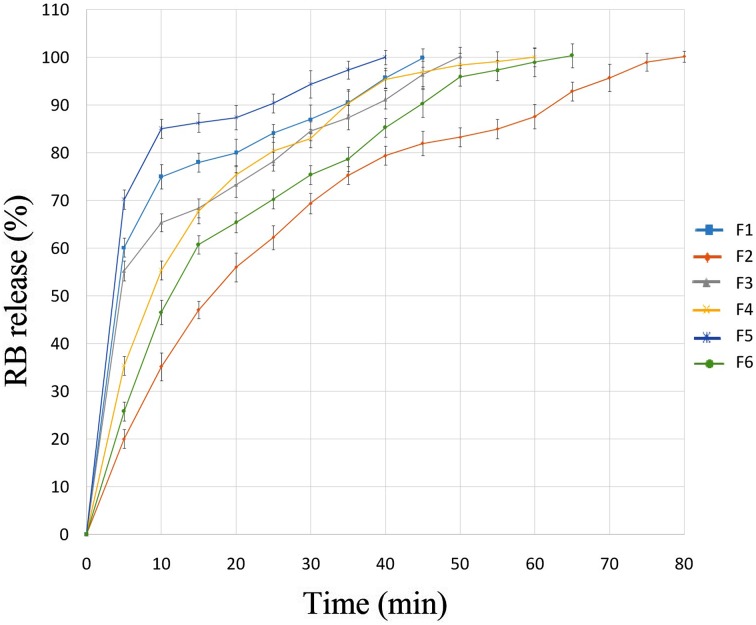
In vitro RB release profile for six buccal film formulations

The in vitro release profile of RB for all formulations is shown in Fig. 10. Results show that RB released from all formulations between 40 and 80 min. The F1, F3, and F5 formulations possessed burst release of RB within first 10 min and gradually increased afterwards. Burst release in mucoadhesive buccal films could depend on swelling ratio. Lower the swelling ratio causes more burst release in the system (El-Samaligy et al. 2004; Patel et al. 2007). For F2, F4, and F6 formulations, RB gradually released at 60–80 min. F2 formulation containing higher concentration of HPMC K4M showed slower release rate compared to the other formulations. Higher HPMC content in films causes more swelling ratio which increases film thickness. Therefore, HPMC K4M layer can reduce drug diffusion through other polymeric material which was also confirmed by other groups working with HPMC-based buccal films (Kumria et al. 2016; Shanker et al. 2009).

### Release mechanism

The mechanism of in vitro release was determined by different equations and kinetic models to explain the release kinetics of RB from buccal films. The kinetic profiles of RB release in PBS were followed by Korsmeyer–Peppas and Higuchi model. *R* ^2^ values were determined by release profile of each model. The release mechanism of RB from all formulations is shown in Table 4. F2, F4, and F6 formulations follow Korsmeyer–Peppas model below 60% drug released and ‘*n*’ values are 0.7472, 0.5967 and 0.7862, respectively. In this case, n values are between 0.5 and 1 and represent the anomalous transport mechanism (non-Fickian diffusion). Meaning that drug is released by diffusion-based and swelling-based mechanism (chain relaxation) simultaneously. These results were also confirmed by another group in previous work (Bahri-Najafi et al. 2014). F1, F3, and F5 formulations follow Higuchi model, which express burst release of almost half of the drug.

**Table 4:** RB release kinetic models and their parameters for different film formulations

Formulations	Zero order		First order		Higuchi		Korsmeyer–Peppas		
	*K*_0_	*R* ^2^	*K*_1_	*R* ^2^	*K*_H_	*R* ^2^	*K*_P_	*n*	*R* ^2^
	(min^−1^)	–	(min^−1^)	–	(min^−1/2^)	–	(min^−n^)	–	–
F1	0.0843	0.9371	0.0105	0.8968	0.7816	0.9624	–	–	–
F2	0.0935	0.9028	0.0158	0.7470	1.1372	0.9741	0.0613	0.7472	0.9968
F3	0.0950	0.9881	0.0123	0.9651	0.9093	0.9909	–	–	–
F4	0.1034	0.8523	0.0148	0.7431	1.1214	0.9413	0.1368	0.5967	0.9958
F5	0.0708	0.8766	0.0082	0.8394	0.6327	0.9222	–	–	–
F6	0.1096	0.9058	0.0171	0.7595	1.2122	0.9706	0.0738	0.7862	0.9956

## Conclusion

Mucoadhesive buccal films of RB were successfully prepared by solvent casting method using different materials including RB, HPMC, PVA, PEO, glycerol, and stevia. FTIR spectra and DSC thermograms showed good compatibility between RB and other components of film formulation. In addition, SEM images confirmed uniform distribution of RB in films. In vitro drug release profiles, disintegration and dissolution time, swelling properties, mechanical properties, and mucoadhesive characteristics of RB-loaded films were investigated. F1, F3, and F5 formulations had burst release effect and they could be applicable for treatment of pain phase of migraine. Among these formulations, F1 formulation had suitable disintegration and dissolution rate along with appropriate mechanical properties. In addition, it showed about 99.89% RB released in 45 min and ex vivo residence time was observed in 13 min when film started to disintegrate and loose shape. On the other hand, F2, F4, and F6 formulations have shown more sustained delivery mechanism compared to other formulations. F2 formulation had the highest tensile strength, ex vivo mucoadhesive strength, and swelling percentage, which is 12.005 MPa, 0.790 N, and 30%, respectively. Therefore, it can be a prominent option in extended release of RB for treatment of aura or prodrome phase of migraine. It also showed about 100.16% RB released in 80 min. Among F1 and F2 formulations, F1 was selected as an optimized formulation because of complete release in lower time. In the present work, we focused on preparing new different formulations of mucoadhesive buccal films which could release 10 mg of RB in 40–80 min along with good mucoadhesive strength. These films were developed and characterized with different physical and mechanical parameters. In the next step, we suggest that ex vivo permeation test along with in vivo study in future will confirm these in vitro results. Overall, this research can be an innovative and promising work for RB delivery in migraine treatment.

## Abbreviations

DSC: Differential scanning calorimetry
EB: Elongation-at-break
FTIR: Fourier transform infrared spectroscopy
HPMC: Hydroxypropyl methylcellulose
PBS: Phosphate buffer saline
PEO: Polyethylene oxide
PVA: Polyvinyl alcohol
RB: Rizatriptan benzoate
SEM: Scanning electron microscope
